# Improving national pharmaceutical supply management in Liberia through strengthening the training of pharmacists

**DOI:** 10.1186/2052-3211-7-S1-O21

**Published:** 2014-12-17

**Authors:** Lloyd Matowe, Jacob Kolawole

**Affiliations:** 1 Pharmaceutical Systems Africa, Monrovia, Liberia; 2 School of Pharmacy, University of Liberia, Monrovia, Liberia

## Background

Under the Global Fund round 8 Grant, the Liberian Ministry of Health and Social Welfare (MoHSW) received funding for Health Systems Strengthening (HSS). This grant sought to contribute to scaling-up efforts to reduce morbidity and mortality associated with HIV/AIDS, TB, malaria and related diseases. The overall goal was to address the health manpower needs at all levels of the healthcare delivery system with improving the quality of curricula for the training of health manpower and standardizing it to conform to international standards as one of the objectives. As a sub-recipient to this grant Pharmaceutical Systems Africa (PSA) worked with local partners to develop a new curriculum for pharmacy at the School of Pharmacy in Liberia and to employ key staff for the School of Pharmacy.

## Method

Working with international partners, PSA used consensus approaches to review existing modules in the pharmacy curriculum and used international experts to develop new contemporary modules. To finalize the curriculum review process an international expert was brought to Liberia to work on the process. To implement the new curriculum an experienced Dean was hired to head the School of Pharmacy in Liberia for a period of two years. To sustain the gains attained during this program, junior members of staff in the University were sent on postgraduate studies.

## Results

A new curriculum for pre-service pharmacy and a new Dean are in place. The new curriculum has a complete semester module on supply chain management. In addition to this supply chain module, another semester is set aside for experiential learning programs on practice sites. Forty percent of this rotational placement is dedicated to supply chain experience. This includes spending time with the Central Medical Stores, the Supply Chain Management Unit, among other supply chain functionaries. In the final year of the program students spend half a semester on further clerkships that among other things seek to buttress their supply chain and clinical skills.

## Discussion

A multi-pronged approach to strengthen the training of pharmacists in Liberia demonstrated that change is possible even in countries emerging from conflicts. The effect of the war in Liberia had resulted in battered health and education system with limited functionality. The pharmacy curriculum in Liberia before this intervention was a ten-page document with limited content. More importantly, none of the lecturers held any post graduate courses of repute or worked in the university full time, making the program a part time professional course. Our interventions succeeded in bring the training of pharmacists in Liberia close to regional levels, such as seen in neighbouring Ghana or Nigeria.

## Lessons learned

Regional and local efforts, if adequately supported, can result in effective system changes. Currently three international partners who have employed our students in supply chain management roles have expressed satisfaction in the level of supply chain competences our students graduate with. Still, we have only graduated one stream based on the new curriculum and much more effort is needed to sustain the gains attained.

